# Entropy and Non-Equilibrium Statistical Mechanics

**DOI:** 10.3390/e22050507

**Published:** 2020-04-29

**Authors:** Róbert Kovács, Antonio M. Scarfone, Sumiyoshi Abe

**Affiliations:** 1 Department of Energy Engineering, Faculty of Mechanical Engineering, Budapest University of Technology and Economics, 1111 Budapest, Hungary; 2 Department of Theoretical Physics, Wigner Research Centre for Physics, Konkoly-Thege M. 29-33, 1121 Budapest, Hungary; 3 Montavid Thermodynamic Research Group, 1112 Budapest, Hungary; 4 Istituto dei Sistemi Complessi, Consiglio Nazionale delle Ricerche (ISC-CNR), c/o, Politecnico di Torino, Corso Duca degli Abruzzi 24, I-10129 Torino, Italy; antonio.scarfone@polito.it; 5 Physics Division, College of Information Science and Engineering, Huaqiao University, Xiamen 361021, China; suabe@sf6.so-net.ne.jp; 6 Institute of Physics, Kazan Federal University, 420008 Kazan, Russia; 7 Department of Natural and Mathematical Sciences, Turin Polytechnic University in Tashkent, Tashkent 100095, Uzbekistan; 8 ESIEA, 9 Rue Vesale, 75005 Paris, France

**Keywords:** non-equilibrium phenomena, kinetic theory, second law of thermodynamics, statistical distributions, stochastic processes

The present Special Issue, ‘Entropy and Non-Equilibrium Statistical Mechanics’, consists of seven original research papers. Although the issue has a long history, still it remains as one of the most fundamental subjects in physics. These seven papers actually cover various latest relevant topics, ranging from gravity as an entropic force to exotic statistics, including conservation laws, dynamics generated by entropy production, quantum measurements and the limits on the constitutive laws in classical gaseous systems.

We hope that the Special Issue will be able to play a role in further progress to come in the future.

In this paper, ‘Classical Model of Quons’, by G. Kaniadakis and A. M Scarfone [1] by using a kinetic interaction principle an evolution equation describing quons statistics is proposed by properly generalizing the inclusion/exclusion principle of standard boson and fermion statistics. In this way, a nonlinear Fokker-Planck equation for quons particles of type I and type II is introduced and the corresponding steady distribution is derived.

The paper ‘Statistics of the Bifurcation in Quantum Measurement’, by K.-E. Eriksson and K. Lindgren [2], deals with a quantum measurement of a two-level system improving the already known methods, basing the analysis of the interaction with the measurement device on the quantum field theory. In this way, a microscopic details of the measurement apparatus affect the process so that it takes the eigenstates of the measured observable by recording the corresponding measurement result.

The paper written by R. Kovács [3] deals with the experiments on rarefied gases. The generalized system of Navier-Stokes-Fourier equations is presented, which is required to model the ballistic effects appearing in gases at low pressures. The experimental evaluation consists of the investigation of scaling properties of models originating from the kinetic theory and continuum thermodynamics, especially emphasizing the importance of mass density dependence of material properties.

In the paper ‘A Note on the Entropy Force in Kinetic Theory and Black Holes’ by R. A. Treumann and W. Baumjohann [4] is derived a kinetic equations of a large system of particles including a collective integral term to the Klimontovich equation for the evolution of the signle-particle distribution function. The integral character of this equation transforms the basic signle particle kinetics into an integro-differential equation showing that not only the microscopic forces but the hole system gets its probability distribution in a holistic way.

In their paper [5], motivated by the contact geometric structure in thermodynamics, V. Klika, M. Pavelka, P. Vágner, and M. Grmela present an approach to multilevel modeling based on the recognition that the state variables and their conjugate variables are independent. That procedure is called Dynamic MaxEnt, and demonstrated on various examples from continuum physics such as hyperbolic heat conduction and magnetohydrodynamics.

In the paper ‘A Fourth Order Entropy Stable Scheme for Hyperbolic Conservation Laws’ [6], Xiaohan Cheng presents the development of a numerical procedure with fourth order accuracy in order to solve hyperbolic system of partial differential equations of conservative form, for one-dimensional situations. Here, the novelty is to endow great importance for the entropy balance equation in updating the state variables, thus the numerical compatibility with the second law of thermodynamics is ensured. Its efficiency is demonstrated on several examples, e.g., on shock tubes and on the nonlinear Burgers equation.

In the paper [7], Ou, Yokoi and Abe note a possible diversity of baths in quantum thermodynamics. They discuss the isoenergetic processes in terms of the concept of weak invariants, where the time-dependent Hamiltonian is a weak invariant associated with a relevant master equation. In particular, they analyze as an explicit example the finite-time isoenergetic process of the nonequilibrium dissipative system of the Pauli spin in a varying external magnetic field based on the Lindblad master equation.
